# Functional connectivity estimation over large networks at cellular resolution based on electrophysiological recordings and structural prior

**DOI:** 10.3389/fnana.2014.00137

**Published:** 2014-11-20

**Authors:** Simona Ullo, Thierry R. Nieus, Diego Sona, Alessandro Maccione, Luca Berdondini, Vittorio Murino

**Affiliations:** ^1^Department of Pattern Analysis and Computer Vision, Istituto Italiano di TecnologiaGenova, Italy; ^2^Department of Neuroscience and Brain Technologies, Istituto Italiano di TecnologiaGenova, Italy; ^3^Department of Computer Science, University of VeronaVerona, Italy

**Keywords:** connectomics, structural connectivity, functional connectivity, high-density Micro Electrode Array, electrophysiology, graph heat kernel, probabilistic directional feature, Von Mises distribution

## Abstract

Despite many structural and functional aspects of the brain organization have been extensively studied in neuroscience, we are still far from a clear understanding of the intricate structure-function interactions occurring in the multi-layered brain architecture, where billions of different neurons are involved. Although structure and function can individually convey a large amount of information, only a combined study of these two aspects can probably shade light on how brain circuits develop and operate at the cellular scale. Here, we propose a novel approach for refining functional connectivity estimates within neuronal networks using the structural connectivity as prior. This is done at the mesoscale, dealing with thousands of neurons while reaching, at the microscale, an unprecedented cellular resolution. The High-Density Micro Electrode Array (HD-MEA) technology, combined with fluorescence microscopy, offers the unique opportunity to acquire structural and functional data from large neuronal cultures approaching the granularity of the single cell. In this work, an advanced method based on probabilistic directional features and heat propagation is introduced to estimate the structural connectivity from the fluorescence image while functional connectivity graphs are obtained from the cross-correlation analysis of the spiking activity. Structural and functional information are then integrated by reweighting the functional connectivity graph based on the structural prior. Results show that the resulting functional connectivity estimates are more coherent with the network topology, as compared to standard measures purely based on cross-correlations and spatio-temporal filters. We finally use the obtained results to gain some insights on which features of the functional activity are more relevant to characterize actual neuronal interactions.

## 1. Introduction

Brain processing is widely recognized to be distributed over a wide range of different scales, involving an impressive number of cells with heterogeneous phenotypes that are structurally and functionally organized in a sophisticated and still unclear architecture. Disentangling the intricate contributions of single neurons constituting large brain circuits from the strongly correlated phenomena shaping brain function is one of the biggest challenges in neuroscience. To complicate things further, most of the neuronal processing taking place in the nervous system is characterized by a limited observability and still requires the additional improvement of currently existing neurotechnologies. Indeed, while direct measurements are only possible at very small scales (i.e., monitoring the intracellular potential of a few single neurons or up to a few hundreds of neurons with 2-photon microscopy), larger scale mechanisms can commonly be observed through indirect non-invasive modalities (i.e., brain imaging) but rather loosing the resolution of single cells. Given these two opposite experimental approaches that have characterized the neuroscientific research over the last decades, what still remains unanswered is how to bridge the structural and functional aspects observed at the different scales.

In the last few decades efforts have been put forward for the investigation of the so-called *connectome*, i.e., the reconstruction of the neural connectivity at different scales (Sporns et al., 2005; Leergaard et al., 2012). The term *connectomics* has a very broad scope, ranging from single-neuron interplays (*microscale* connectomics) to pathways between large brain regions (*macroscale* connectomics, Yap et al., 2010). Reconstructing the brain connectome across these scales is fundamentally important to understand the constituent parts of the nervous system, their multiple interactions and the advanced cognitive functions that they support, both in normal and pathological neurodegenerative conditions. By promoting the analysis of different aspects of brain behavior, connectomic studies typically involve two complementary forms of information: structure and function.

In the literature these two aspects are usually studied separately. Part of the efforts focuses on a dense reconstruction of the *structural connectivity*, while complementary studies address the analysis of synchronous patterns of neuronal activation for estimating the *functional connectivity*.

However, structure and function are tightly interrelated. By looking at fine-scale interactions, we are learning that the functional properties of single neurons are strongly driven by their anatomical interconnections with other cells, dendritic arborizations, and synaptic distributions. At the same time, single-neuron physical links affect the expression of functional patterns throughout the entire network by placing constraints on which functional interactions are more likely to occur. Consequently, it is getting crucial to combine a detailed description of the anatomical connectivity patterns with physiological parameters to capture the way functional properties emerge from structural configurations at the cellular scale.

This work addresses this challenge by proposing a combined structural and functional analysis of large neuronal networks that are functionally resolved at an unprecedented resolution, approaching the scale of single-neurons.

The joint study of structure and function has been recently gaining interest in the context of brain imaging modalities (Rykhlevskaia et al., 2008), where it is possible to observe large-scale interactions. Recent attempts address the estimation of functional connectivity guided by the structural connectivity as prior (Deligianni et al., 2011; Chen et al., 2013; Zhu et al., 2013). The underlying hypothesis is that the functional connectivity must reflect the existence of structural paths connecting functionally linked regions (Honey et al., 2010). However, *macroscale* approaches are not suitable for single-neuron resolution as they deal with large areas (billions of neurons) that make any fine-grained analysis unfeasible.

On the other hand, *microscale* connectomics achieves good resolution by focusing on single or few cells, but looses the information on network-wide topology and interplays. A new branch of investigation is recently emerging studying the so-called *mesoscale* that, in principle, could overcome the limitations of *micro* and *macro* studies. Mesoscale connectomics refers to the analysis of connectivity at the level of neuronal circuits with a micrometric spatial resolution (Sporns, 2012). Interestingly, high-level functions such as learning and memory build on stratified non-linear mechanisms that can be particularly witnessed at this scale (Jimbo et al., 1999; Marom and Eytan, 2005). Although there is still no clear indication about the possibility of bridging the gap between the different scales at which the brain is currently investigated, there are studies highlighting the role of specific neurons (hub neurons) in determining emergent network dynamics (Bonifazi et al., 2009).

Thanks to recent technological advances, it is nowadays possible to collect high-resolution structural and functional information at the mesoscale from cultured neuronal networks. This enables the development of new methodologies for a combined structural and functional analysis at this scale. In particular, novel generations of active Micro Electrode Arrays (MEAs), such as the High-Density MEA (HD-MEA) chips introduced by Berdondini et al. (2009), allow to record the electrical activity of neuronal networks from thousands of electrodes at sub-millisecond resolution and at the granularity of the single cell. The combination of such a high-resolution functional data with fluorescence microscopy imaging can enable the unprecedented mapping of both activity and structure of neural assemblies at a cellular level. Indeed, relatively sparse neuronal cultures–grown on-chip by seeding few thousand cells–allow to acquire detailed spatio-temporal recording of neuronal activity and topographic distribution of neurons with respect to the electrode array. This provides the unique chance of correlating functional activity with neuronal topology over large assemblies.

This work proposes a computational framework for the joint analysis of functional and structural connectivity at the mesoscale which takes advantage of the remarkable spatial resolution offered by HD-MEAs.

In particular, we start from the reasonable hypothesis that the presence of a strong structural connection makes a functional connection more likely to occur. The influence of the network topology on the functional behavior has been already proven on a theoretical level (Kriener et al., 2009). Furthermore, distance and strength of cross-correlation have been proven to be related also *in vivo* (Hirase et al., 2001) and *in vitro* (Shlens et al., 2006). However, experimental studies at neuronal resolution covering large networks are typically more difficult to carry out due to both technological constraints and problem complexity. Here, we address this task by developing a set of computational algorithms that enables the combined structural and functional analysis of networks with thousands of neurons.

This could not be done on conventional MEAs that typically integrate 60–256 microelectrodes, and where existing studies are typically limited to the analysis of network-wide electrophysiological activity. Consequently, the absence of any anatomical evidence to support functional hypotheses strongly limits the potentiality of this analysis. Few recent attempts have been presented in literature addressing multimodal studies at the mesoscale. Abdoun et al. (2011) introduced the NeuroMap software tool for handling MEA recordings co-registered with fluorescence images. However, in this tool, the image is used only for visualization purposes. Another multimodal study has been proposed by Becchetti et al. (2012) for differentiating the functional activity of excitatory and inhibitory neurons from MEA recordings and GAD67-GFP imaging. Their method is based on a manual extraction of the structural information (i.e., visually classifying excitatory from inhibitory cells) lacking in any further characterization of the network anatomy (e.g., the topology). As no structural connectivity information is available, the assessed statistical properties of the electrophysiological signals only account for local functional dynamics, discarding more complex network interactions. Furthermore, in both cases the use of standard MEAs (up to few hundred electrodes) offers poor spatial resolution. Unlike HD-MEAs, these systems cannot provide the possibility of monitoring both single-cell activity and wide network dynamics at the same time.

In this paper, we propose a framework for integrating multimodal information acquired on HD-MEAs with the aim of refining the estimate of functional connectivity using the structural connectivity as prior. Specifically, we localize neurons with respect to the electrode array and estimate the structural connectivity of the electrodes to compute the topological distance along the paths connecting them. This is used as structural information to refine a rough estimate of functional connectivity based only on cross-correlations. As extensively suggested in the literature (Feldt et al., 2011), graph theory is used to support the analysis by describing the network connectivity with graph representations. Neuronal networks perfectly fit into this framework as it provides the flexibility to characterize both structure and function from anatomical and electrophysiological observations (Bullmore and Sporns, 2009).

**Figure 1:**
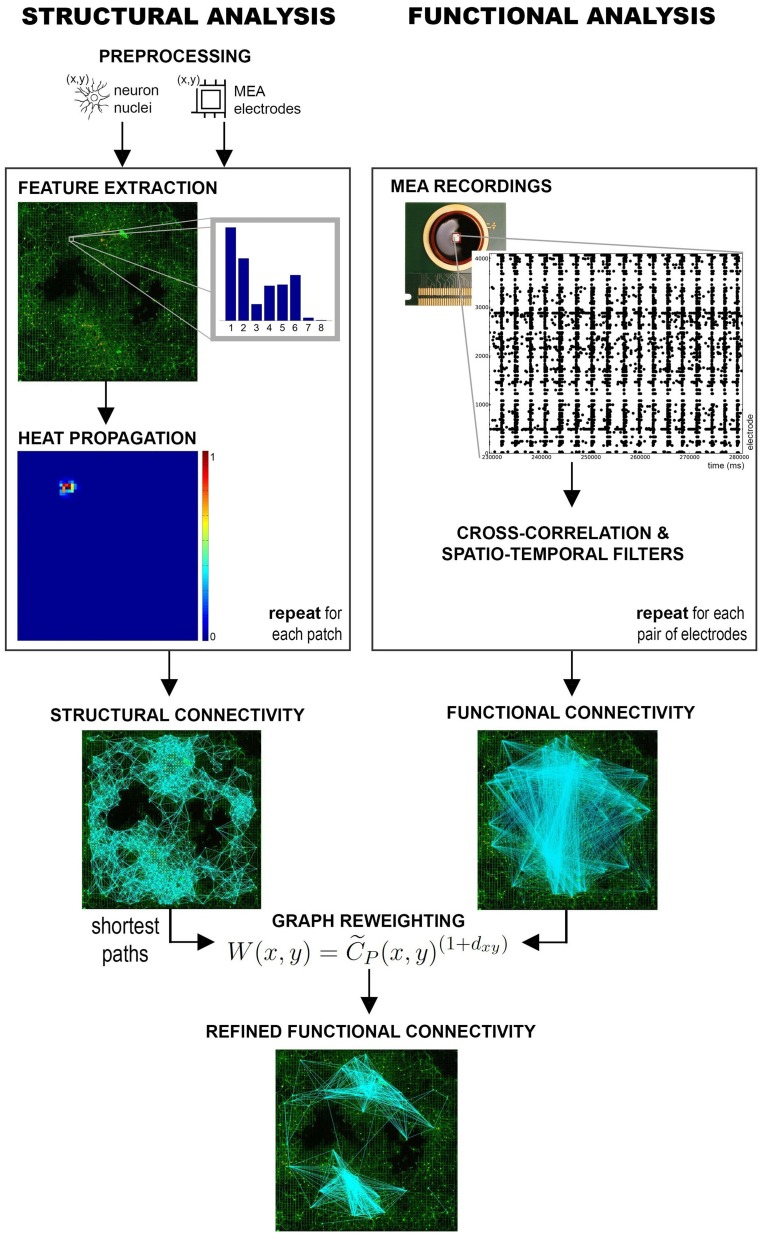
**Overview of the approach**. Structural and functional connectivity maps are separately estimated from the multimodal datasets acquired on HD-MEAs. The functional graph is then refined using the structural information as prior.

An overview of the proposed approach is provided in Figure 1. Structural connectivity maps are first estimated from fluorescence images of the neuronal culture by using local directional features and heat propagation (Ullo et al., 2013). The obtained prior on the existing anatomical links is then used to refine the estimate of functional connectivity which is obtained from cross-correlation measures of the electrophysiological signals, as introduced by Maccione et al. (2012). In this fashion, the anatomical information offers a reference space facilitating the interpretation of the observed functional interactions.

The contributions of this paper are twofold. First, we introduce a computational framework capable of estimating the structural connectivity of large neuronal assemblies and we show how more reliable estimates of functional connectivity can be obtained by incorporating such structural information as prior. Second, we use the obtained results to formulate new hypotheses on relevant features of the electrophysiological activity that can better characterize functional interactions between neurons.

## 2. Materials and methods

### 2.1. Electrophysiological recordings and cell culture staining

Cell cultures were recorded by means of High-Density Micro Electrode Arrays (HD-MEAs). These commercially available devices (www.3brain.com), have been extensively described in Imfeld et al. (2008) and Berdondini et al. (2009). Briefly, high-density MEAs allow simultaneous extracellular recordings from 4096 square electrodes (pitch = 42 μm) arranged in a 64 × 64 layout (2.7 by 2.7 mm^2^ active area) at a sampling rate of about 7 kHz per channel.

Primary hippocampal neurons from rat embryos at E18 were dissociated by enzymatic digestion and seeded on HD-MEAs previously sterilized and coated with polylisine adhesion factor (Maccione et al., 2010, 2012). Drops of 30–50 μL were seeded over the active area of the chip at a nominal low concentration of 100–150 cell/μL. After 2–3 weeks in incubator, cultures develop a sparse interconnected network structure showing synchronous functional activity. Extracellular electrophysiological recordings of neuronal signals were acquired at 18–19 Days *In Vitro*. Spontaneous activity was recorded for 10–15 min as control condition, followed by another 10–15 min recording under chemical stimulation by adding 30 μMol Bicucculline.

After electrophysiological recordings, neuronal tissues were fixed on the chip array in 4% paraformaldehyde for 20 min and stained with NeuN for neuronal nuclei and β3-tubulin for axonal and dendritic arborization (Maccione et al., 2012). Cultures were then inspected under a microscope, collecting multiple fields at 20× magnification with a micro positioning stage. The acquired portions were then stitched together using Adobe Photoshop CS3 and the open source free software Fiji (http://fiji.sc/Fiji).

### 2.2. Multimodal dataset description

The combination of the HD-MEA technology with the immunofluorescence microscopy results in multimodal datasets, each consisting of a high-resolution fluorescence image—i.e., the *structural* data—and a set of electrophysiological recordings—i.e., the *functional* data^1^.

For the purpose of our experiments, two different neuronal networks were cultured on HD-MEAs under the same experimental conditions. **Figure 5A** shows the fluorescence images of the two cultures. In each neuronal culture about one thousand of cells were grown, showing a strong degree of structural connectivity. As we aim at investigating the excitatory functional connectivity, we focus on the analysis of the electrophysiological recordings with added Bicucculline, a blocker of the inhibitory pathway. This choice limits the number of potential inhibitory connections and is a desirable condition since the cross-correlation (as defined by Equation 3) is only designed to detect excitatory functional connections (Garofalo et al., 2009). The raw electrical signal recorded by each electrode was encoded, after spike detection (Maccione et al., 2009), as a sparse vector of size *f*_*s*_ × *t*_*r*_, where *f*_*s*_ is the sampling frequency and *t*_*r*_ is the recording time interval. The whole network recording is arranged in a sparse matrix, where the indexing (*i*, *j*) refers to the electrode at row *i* and column *j* in the electrode array. Each electrode (*i*, *j*) is then associated to a vector with the time stamps of the corresponding spiking activities. This encoding of the spiking activity is used as input data for estimating the functional connectivity (see Section 2.4).

The presented cases of study were selected as representative in terms of number of neurons, density of connections and number of functionally-correlated signals. Detailed information on each dataset are reported in Table 1. First order statistics on cell culture dynamics are in line with previous studies (Maccione et al., 2012).

**Table 1:** **HD-MEA dataset description**.

**Dataset**	**Image size**	**# Active electrodes**	**MFR (Hz)**	**MBR (#burst/min)**	**MFIB (Hz)**	**MBD (ms)**
Chip-253	2985 × 2982	146	1.63 ± 0.12	8.76 ± 0.06	71.23 ± 0.25	122.80 ± 0.60
Chip-250	2928 × 2946	181	0.77 ± 0.09	4.08 ± 0.04	59.82 ± 0.29	150.38 ± 0.88

### 2.3. Structural connectivity analysis from fluorescence images

Dissociated neuronal cultures show extensive and fuzzy connectivity that makes structural analysis computationally hard. To tackle this challenge, a method based on heat propagation is used to estimate the structural connectivity of neuronal assemblies with dense connectivity, as reported in Ullo et al. (2013).

The method provides a description of the network topology in terms of a graph where nodes correspond to the electrodes and edges represent structural connections. In fact, this provides the common reference frame to relate the functional signal recorded by the HD-MEA to the network anatomy.

Maps of electrode connectivity are determined using a Graph Heat Kernel (GHK) framework (Belkin and Niyogi, 2003; Bai et al., 2010) based on probabilistic directional features (Ullo et al., 2013). These features encode the local directionality of the neurites within small patches of the image corresponding to the MEA electrodes. A feature consists in a histogram with 8 entries, each representing the probability of the current electrode being connected to each of its adjacent neighbors (both horizontal, vertical, and diagonal adjacency are considered).

**Figure 2:**
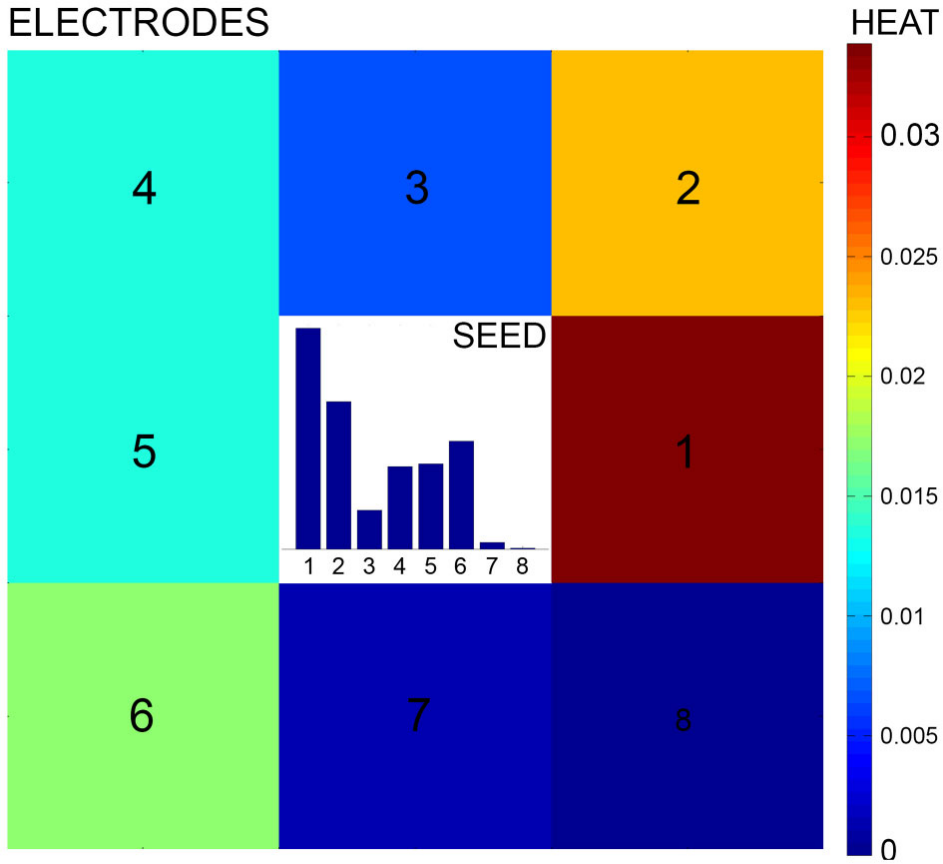
**Heat propagation**. A heat source is placed at the seed electrode and propagated according to the probability of connection defined by the directional feature. Heat propagation favors directions with higher probability of connection. The adjacent electrodes (numbered from 1 to 8) are reached by a different amount of heat according to the seed feature, as described by the colormap.

In its general formulation, a GHK allows to estimate the structure of a graph by computing the amount of heat that propagates from a source to a destination node. The intuition behind the use of a GHK for structural connectivity estimation can be explained by first considering the lattice formed by the regular MEA structure. A weighted graph can be defined on this lattice where the electrodes are nodes and edge weights are given by their degree of connectivity, i.e., by the values of the corresponding probabilistic directional features (see Figure 2). If we placed a certain amount of heat on a *seed* node and let it propagate through the graph, heat propagation would favor the edges having higher weights, i.e., corresponding, in principle, to stronger connections. As a result, the amount of heat reaching a destination electrode from the seed could be considered as an estimate of the strength of their connectivity. Repeating this propagation for all seed electrodes, we can obtain an estimate of the whole-network structural connectivity. Only electrodes having neurons in their recording area are considered as seeds, as they are the ones substantially contributing to the electrophysiological activity.

Further details on the structural analysis will be provided in the following sections.

#### 2.3.1. Probabilistic directional features.

**Figure 3:**
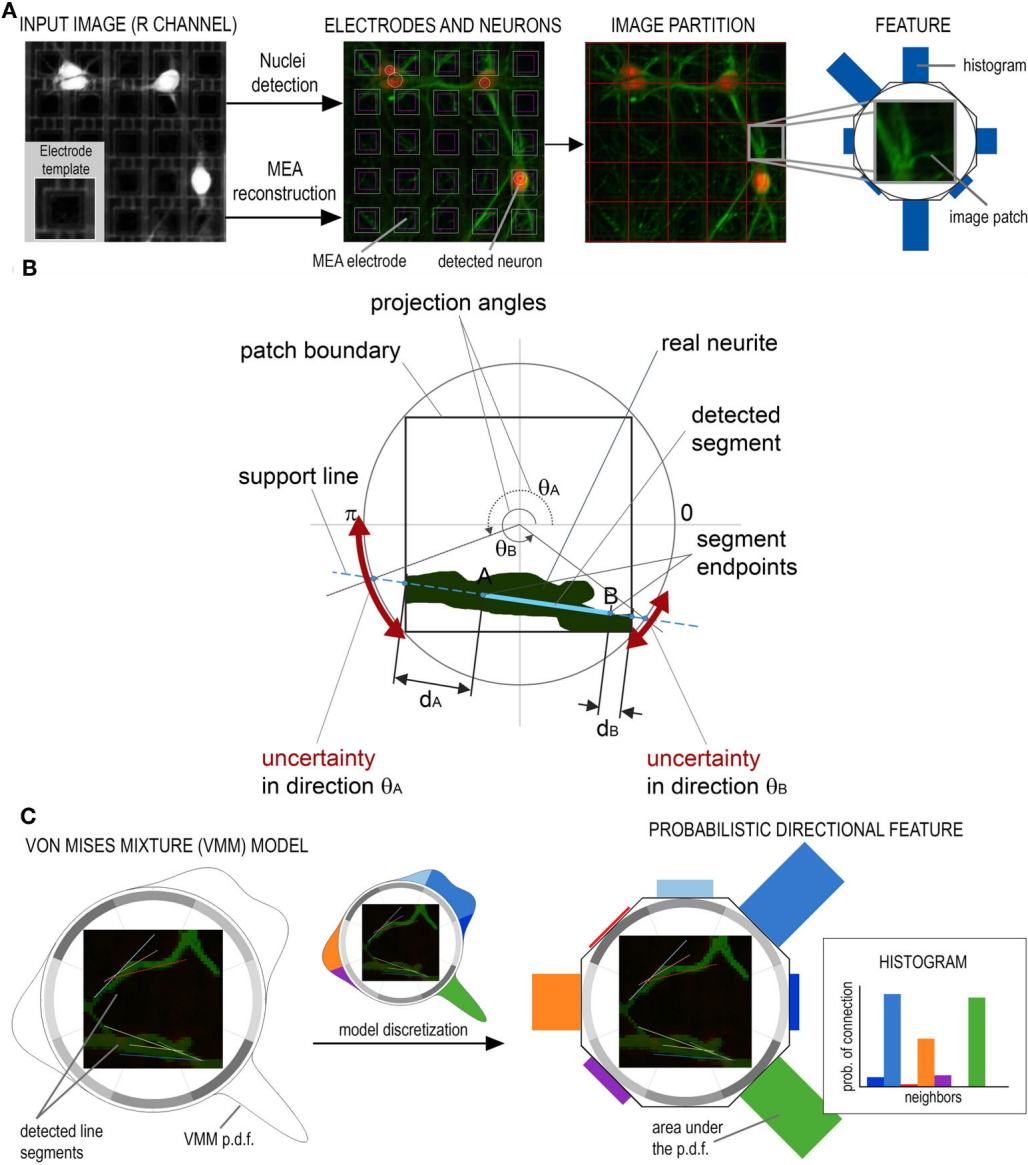
**Preprocessing and feature extraction. (A)** The Micro Electrode Array reconstruction is used to partition the image into small patches corresponding to the electrode areas. **(B)** A Von Mises Mixture (VMM) model is fitted to a set of segments detected on the patch to describe the uncertainty of local neurite orientation. **(C)** The VMM is then discretized to obtain the feature, i.e., an histogram describing the probability of connection with adjacent electrodes.

A preprocessing pipeline is first run to detect neuronal nuclei and reconstruct the electrode array from the image as reported in Ullo et al. (2012). Specifically, the MEA reconstruction allows to compute an electrode-based partition of the image, i.e., a partition into small patches corresponding to the electrode areas (see Figure 3A). The proposed directional features are then extracted from each patch with the aim of obtaining the probability of connection between neighboring electrodes as explained by Figures 3B,C. The features characterize the local configuration of neurites' orientations using a directional Von Mises Mixture (VMM) model fitted to a number of line segments.

Von Mises distributions are widely used to describe directional statistics on the circle (Mardia and Jupp, 2000) and are defined by two parameters: mean μ and concentration κ. The larger is the value of the concentration, the higher is the clustering of the points around the mode placed at θ = μ.

In our framework, segments—approximating real neurites—are detected at each image patch using the Hough Transform. A different Von Mises distribution is then fitted to each of the segment endpoints. The goal is to describe the main neurite orientations inside the patch and the corresponding uncertainty in each of the given directions. Uncertainty is associated to the angles at which a neurite exits/enters the patch and is due to the approximation of real neurites by line segments (which can be affected by errors caused by noise, blurring, etc.). To fit the parameters of the Von Mises (VM) distribution, we compute the angle θ_*A*_ (θ_*B*_) as the projection of the segment endpoint *A* (*B*) onto the circle circumscribing the patch, as shown in Figure 3B. The angle defines the mean μ_*A*_ (μ_*B*_) of the VM fitted at endpoint *A* (*B*) which represents the most probable angle at which the neurite enters/exits the patch. To model the uncertainty of this orientation we compute the distance *d*_*A*_ (*d*_*B*_) between endpoint *A* (*B*) and the boundary of the patch. The higher this distance, the higher the uncertainty of the neurite crossing the boundary exactly at the estimated angle. Consequently, the concentration parameter κ_*A*_ (κ_*B*_) is set as inversely proportional to distance *d*_*A*_ (*d*_*B*_).

For a patch with *n* segments, 2*n* VM distributions will be fitted to the data and used to define the VMM model. As the Hough Transform assigns each segment a vote depending on its evidence on the image, votes are used to define mixture proportions. As a results, segments having stronger evidence will be assigned higher weight in the mixture model. An example of VMM model is shown in Figure 3C.

Finally, the obtained probability distribution is discretized in the 8 neighboring directions. This is done by computing the area under the probability density function of the VMM model in 8 different sectors of the circle, as shown in Figure 3C. This results in a histogram in which each entry represents the probability of the current electrode being connected to its neighbors.

#### 2.3.2. Graph heat kernel

The heat kernel specifies how the information flows across a network or a manifold in time. Generally speaking, the goal of the heat kernel is to reduce the dimensionality of high-dimensional data lying on sub-manifolds, so it is related to the concept of spectral clustering (Luxburg, 2007). Similarly, it can be used to geometrically characterize the structure of a graph residing on a manifold by defining its pattern of geodesic distances (Bai et al., 2010).

Given a weighted graph *G* = (*V*, *E*, *W*), where *V* is the set of nodes, *E* ⊆ *V* × *V* is the set of edges, and *W* the matrix of edge weights, the heat diffusion on *G* is defined by the *heat equation*:

(1) $$\left(L_{G}+\frac{\partial }{\partial t}\right)h_{t}=0;$$

where *h*_*t*_ is the heat distribution at time *t* and *L*_*G*_ is the *Graph Laplacian* operator (Belkin and Niyogi, 2003). In particular, *L*_*G*_ = *D* − *A*, where *A* is the symmetric *adjacency matrix* defined on graph *G*, and *D* is the *diagonal degree* matrix whose diagonal elements are given by *D*(*x*, *y*) = ∑_*y*∈*V*_ *A*(*x*, *y*).

As the time derivative of the kernel is determined by the graph Laplacian, the solution of the heat equation is obtained by exponentiating the Laplacian eigensystem over time. According to spectral graph theory, the *heat kernel* has the following eigendecomposition (Bai et al., 2010):

(2) $$h_{t}(x,y)=\sum_{i\;=\;0}^{\left|V\right|}e^{-\lambda_{i}t}\phi_{i}(x)\phi_{i}(y),$$

where *h*_*t*_(*x*, *y*) is the heat kernel element for nodes *x* and *y*, and λ_*i*_ and ϕ_*i*_ are the *i*th eigenvalue and eigenvector of the Graph Laplacian, respectively.

The heat kernel *h*_*t*_(*x*, *y*) is the solution of the heat equation with heat source placed at point *x* at time *t* = 0, and represents the amount of heat at point *y* after time *t*.

The heat kernel solution is generally computed in two steps: (1) the manifold is approximated by the adjacency graph *A* computed from data points and incorporating neighborhood information, and (2) the weighted graph Laplacian is used to estimate the real manifold, optimally preserving such neighborhood information (Belkin and Niyogi, 2003).

In our application, the weighted adjacency matrix *A* is obtained from the probabilistic directional features, by defining each element *A*(*x*, *y*) as the histogram value for the edge connecting electrode *x* to electrode *y* (defining their probability of being connected by a neurite). Due to the way features are defined, histogram values are not symmetric in the two directions, so the matrix *A* needs to be symmetrized, as requested by the GHK formulation. This is done by summing the probability contributions in the two edge directions.

The output of the heat kernel is a |*V*| × |*V*| adjacency matrix (4096 × 4096 in our case) indicating the electrode connectivity in terms of amount of heat propagated after time *t* from a seed electrode. Matrix weights are normalized (divided by the maximum value in the matrix) to obtain the final structural connectivity map. As a matter of fact, this matrix is quite sparse, as only electrodes having neurons in their recording area are taken into consideration as nodes. This allows to limit the connectivity estimation to actual neurons lying on electrodes that can contribute to the electrical activity recorded by the HD-MEA.

**Figure 4:**
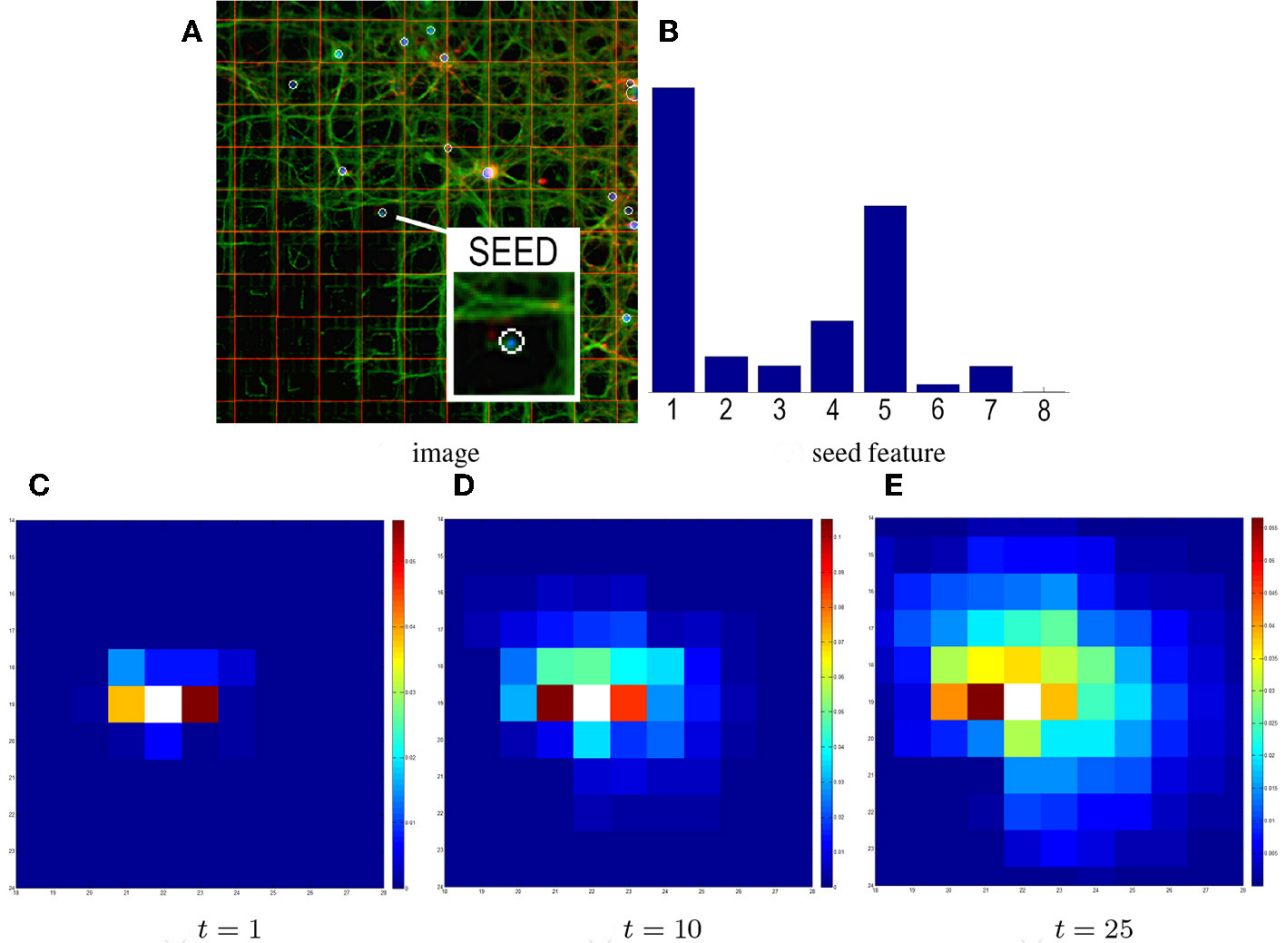
**Influence of time on heat propagation**. The heat propagation time directly influences the estimated connectivity. **(A)** The input image and seed electrode. **(B)** The corresponding probabilistic directional feature. Higher histogram values in bins #1 and #5 reflect the structure of neurites in the patch (spreading horizontally). **(C)** Result of the GHK propagation at time *t* = 1. Electrodes are colored based on the amount of heat propagated from the seed. After time *t* = 1 the seed feature mainly contributes to the propagation, so electrodes 1 and 5 have warmer colors. **(D–E)** Results at *t* = 10, 25. Although the pattern of propagation partially preserves the initial layout, more contributions come from neighboring features.

In the GHK framework, heat propagation is regulated by the time parameter *t*. Figure 4 shows the influence of this parameter on the final estimate. It can be observed that, when *t* grows, a larger portion of the graph is explored, resulting in the overlap of multiple feature contributions (in addition to the initial seed feature). While this makes it more likely to introduce false positives, it also allows to discover new branches in the network connectivity and to compensate for imprecise and noisy local contributions. Hence, setting the value of *t* is a trade-off that strongly depends on the size of the considered domain (in our case the 64 × 64 matrix of electrodes).

### 2.4. Functional connectivity analysis from spiking activity

A cross-correlation based approach for functional connectivity estimation applied to electrophysiological recordings of *in vitro* populations has been recently validated on the HD-MEA recording system in Maccione et al. (2012). Cross-correlations are computed between pairs of electrode signals to obtain a first rough estimate of functional connectivity. For each pair of electrodes (*x*, *y*) (with at least one spike to ensure presence of activity) the following cross-correlation function (*cross-correlogram*) is evaluated among their spike trains:

(3) $$C_{xy}(\tau )=\frac{1}{\sqrt{N_{x}N_{y}}}\sum_{s=1}^{N_{x}}\sum_{t_{i}=\tau -(\Delta_{\tau }/2)}^{\tau +(\Delta_{\tau }/2)}x(t_{s})\;y(t_{s}+t_{i})\;\;$$

with *N*_*x*_ (*N*_*y*_) being the number of spikes in train *x* (*y*), *t*_*s*_ the spike occurrence time in train *x* and Δ_τ_ the time window in which synchronous spikes in train *y* are counted. Δ_τ_ is set at 0.5 ms.

The resulting normalized cross-correlations are then post-processed using a filtering strategy to remove false positives not compatible with biological prior. In particular, the maximum propagation velocity for *in vitro* biological preparations (400 mm/s, Bonifazi et al., 2005) is used to discard physiologically implausible links, i.e., links having correlation peak latency below this value. Such a physiological filter also accounts for delayed spikes in post-synaptic cells. Cross-correlations are then thresholded to retain only statistically significant links. To this aim, the cross-correlation of jittered spike trains [by ±5 ms, thus maintaining the same Inter Spike Interval (ISI) distributions] is computed as null model and a significance threshold *C*_*s*_ is defined using a non-parametric statistical test at *p*-value *p* = 0.05. This shuffling procedure, also called dithering (Grün and Rotter, 2010), is repeated 100 times on each randomly-selected pair of channels. The probability of the jittering was set as uniform in the ±5 ms time interval.

The thresholded functional graph, weighted by the cross-correlation values, relies entirely on the recorded electrophysiology, discarding the valuable information coming from the structural modality.

### 2.5. Combining structural and functional information

We build on the hypothesis that functional co-activation commonly relies on anatomical connections to refine the estimate of Functional Connectivity (FC) from our Structural Connectivity (SC) prior. In order to coherently combine structural and functional information, the refinement process starts from the unthresholded cross-correlation values obtained after spatio-temporal filtering.

As a first step, we observe that the functional connectivity estimates are unaware of the actual neuronal distribution on the array of electrodes. Due to noise affecting the spike detection and/or strong dendritic arborization (whose activity can be, in some cases, detected by the MEA), electrodes with no neuron in their recording area are sometimes included in the graph. As the proposed structural analysis is capable to retrieve a unique correspondence between neurons and electrodes, the first refinement stage consists in discarding such nodes from the FC graph. Additionally, neuronal correlations have been shown to decay with physical distance (Vincent et al., 2013). Nevertheless, cross-correlation measures do not strictly reflect this behavior—due to noise and random co-activations—and the resulting FC graphs frequently present a substantial number of long-range links that are improbable, given the underlying network topology. Thresholding strategies, used to select a subset of somehow relevant links, are typically based on purely empirical observations due to the absence of any ground truth information.

We take advantage of the relationship between functional correlation and structural distance, to define the second step of our refinement strategy, called *reweighting*.

Specifically, the FC values (i.e., normalized cross-correlation peaks) associated to the functional graph are reweighted based on the distance of the corresponding nodes. This measure—called *structural distance*—is the euclidean distance computed along the shortest path connecting the nodes on the structural graph. In principle, we want our algorithm to penalize functional links according to this value. To this aim, a functional link connecting electrodes *x* and *y* with cross-correlation peak defined as:

(4) $$C_{P}(x,y)=\underset{{}_{\tau }}{\max}C_{xy}(\tau ),$$

is reweighted according to the following formula:

(5) $$W(x,y)=\;{\tilde{C}}_{P}{\left(x,y\right)}^{\left(1\;+\;d_{xy}\right)}$$

with *d*_*xy*_ being the structural distance and $\tilde{C}$_*P*_(*x*, *y*) being the cross-correlation peak normalized in the interval [0, 1]. As the distance *d*_*xy*_ is also normalized in the same interval [0, 1], the resulting weights *W* reflect our initial hypothesis.

Finally, a threshold of statistical significance for the estimated functional links is determined by applying the reweighting process to the null model introduced in Section 2.4. A statistical significance test at *p*-value *p* = 0.05 is then used to define a significance threshold *W*_*s*_. As will be discussed in Section 3, results of the refined and thresholded FC graphs show that, by incorporating the structural information as prior, it is possible to provide estimates of functional connectivity more coherent with the network topology.

### 2.6. Classification of functional connections

After the reweighting and thresholding of the initial FC graph, a subset of the original functional links is discarded. We want to investigate if the two classes of discarded and retained connections are characterized by distinguishable functional features. First of all, this would allow to show that the structural prior is not only imposing an *a priori* constraint on the functional connectivity but it is effectively selecting links that behave differently from a functional point of view. Second, this investigation could give some insights on how to effectively detect functional links purely from the analysis of the electrophysiological activity. Former studies (e.g., Ostojic et al., 2009) are informative on how the cross-correlation function is affected by variations of the network background activity, the synaptic strengths and the local network connectivity. In line with these studies, we compute a set of features of the cross-correlogram (i.e., cross-correlation peak, time lag of the peak, spread of the cross-correlation function) that might be informative on the occurrence of actual functional connections. For each discarded/retained link, functional features are computed from the analysis of the original spike trains. As they are not affected by our structural prior, this allows to highlight any intrinsic property of the functional activity capable of revealing real co-activations. The sets of discarded/retained links are then regarded as two classes and a linear Support Vector Machine (SVM) (Duda et al., 2000) is trained/tested on these data to quantify the discriminative power of different combinations of features. The functional features considered in this study are the following:

1. cross-correlation peak (*C*_*P*_)
2. counts/occurrences of the cross-correlation peak (*C*_*O*_)
3. cross-correlation time lag (*C*_τ_)
4. entropy of the cross-correlation function (*C*_*H*_)
5. firing rates of the correlated electrodes (*MFR*_*x*_, *MFR*_*y*_).

Features *C*_*P*_ and *C*_*O*_ are related to the strength of a given functional link, whereas the time lag of the peak (*C*_τ_) will likely be proportional to the closeness of the correlated nodes. The spread of the cross-correlation function can be informative of the nature of a given link: broad functions would likely correspond to unreliable and noisy cross-correlations. As previously shown in Maccione et al. (2012), this feature is also related to the link length. Here, instead of resorting to a gaussian fit of the cross-correlation functions (Maccione et al., 2012), the spread is measured in terms of the more general entropy measure (*C*_*H*_) quantified as

(6) $$C_{H}=-\sum C_{n}(\tau )\;\log_{2}C_{n}(\tau )$$

with *C*_*n*_(τ) = *C*(τ) / ∑_τ_ *C*(τ).

We also included the mean firing rates (*MFR*_*x*_, *MFR*_*y*_) that are typically used to quantify first order statistics of cell culture dynamics. In principle, the firing rates cannot be regarded as effective predictors of any correlated activity, however, at higher firing rates the probability of coincident events (i.e., correlated activities) increases. In addition, since *C*_*O*_ is related to *C*_*P*_ by the geometric mean of *MFR*_*x*_ and *MFR*_*y*_ (by the relation *C*_*O*_ = *C*_*P*_ *T* $\sqrt{MFR_{x}\cdot MFR_{y}}$, with *T* being the length of the recording session), this further motivates the investigation of the interplay between all the features determining the cross-correlation function.

To evaluate the performance of the classifier, we adopt a cross validation (CV) procedure. The original dataset is split into two complementary subsets used, respectively, for training and testing the classifier. Specifically, a standard 10-fold CV is carried out that consists in subdividing the original dataset into ten subsets, the training is performed on 9/10 of them and the accuracy (i.e., performance) of the linear SVM classifier is evaluated on the tenth. This procedure is repeated ten times by alternating the tested subset. The performance of the linear SVM is then quantified as the mean and standard deviation of the accuracies obtained by the 10-fold CV procedure.

## 3. Results and discussion

### 3.1. Functional connectivity estimation from structural prior

**Figure 5:**
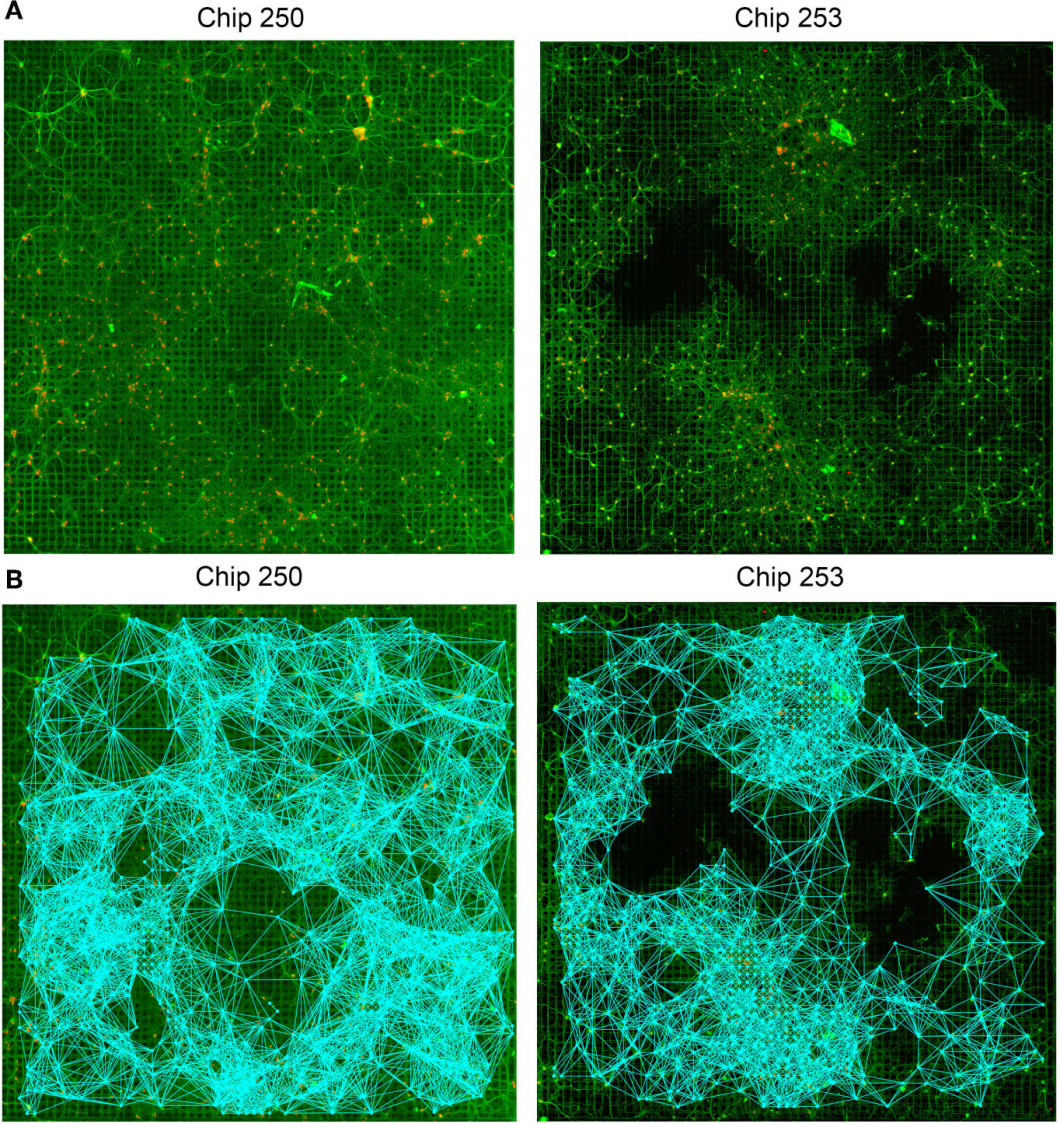
**Structural connectivity graphs. (A)** Original fluorescence images of two neuronal networks (Chip-250 and Chip-253). **(B)** Resulting structural connectivity graphs estimated with the GHK framework. The networks present high degree of connectivity (5570 SC links for Chip-253 and 7808 links for Chip-250).

The proposed approach was applied to the analysis of the two HD-MEA datasets described in Section 2.2. The structural connectivity of the network was first estimated by running the GHK algorithm for each *seed* electrode, i.e., for each electrode having at least one neuron in its recording area. Propagation time was chosen experimentally and set to *t* = 25, taking into account the extent of the domain, i.e., the 64 × 64 initial lattice defined on the MEA structure. The value provides a good trade-off between the capability of the system of exploring the graph and the introduction of spurious connections due to the extended contribution of neighboring features. For further details on the study of the time parameter the reader is referred to Ullo et al. (2013). The estimated SC graphs are shown in Figure 5B and both reflect the strong degree of connectivity of the networks (5570 and 7808 SC links were estimated for Chip-253 and Chip-250, respectively).

**Figure 6:**
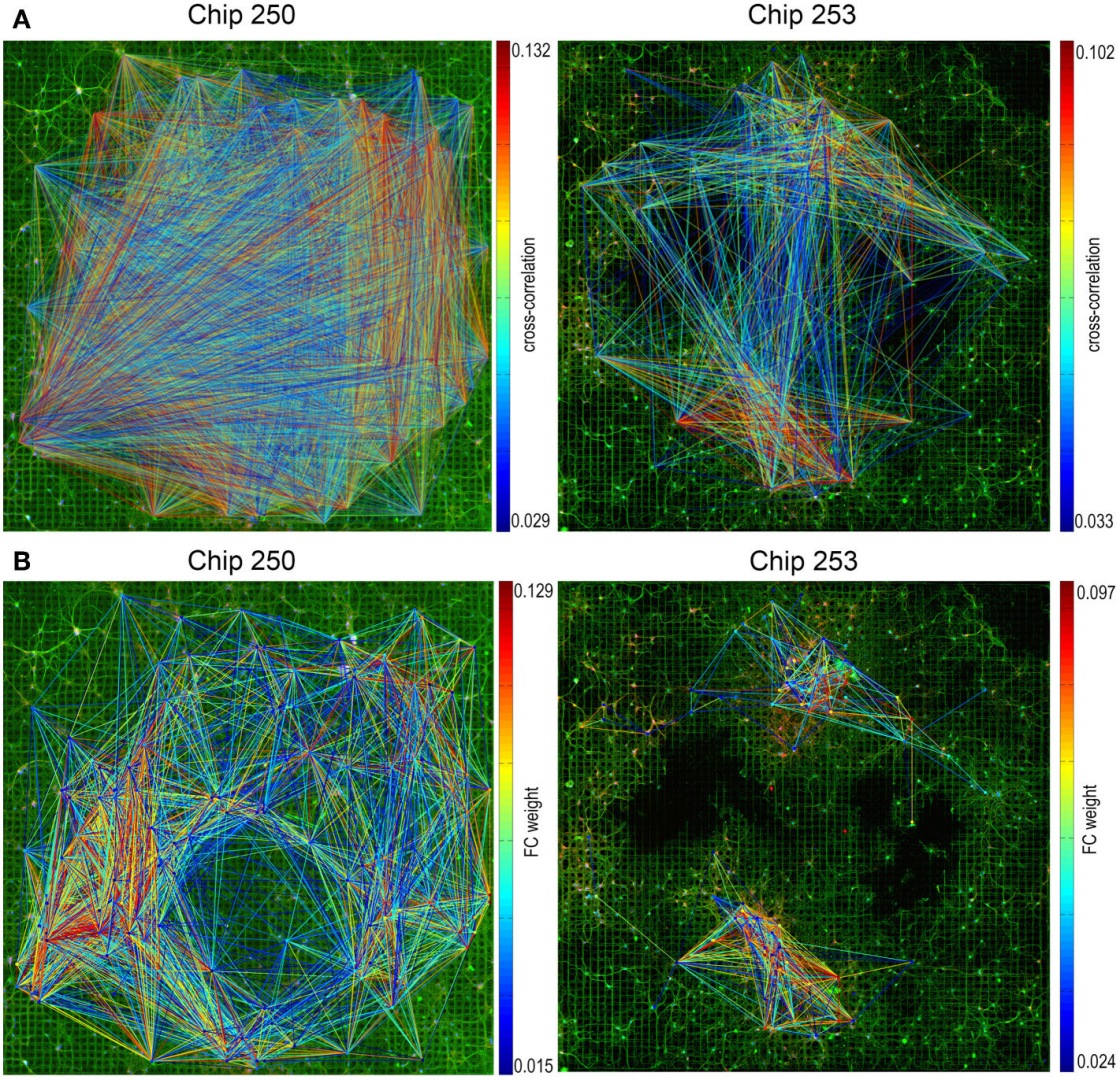
**Functional connectivity graphs. (A)** Functional connectivity graphs estimated computing cross-correlations on the pairwise electrophysiological signals and applying spatio-temporal filters and a significance threshold based on dithering. Functional links are color-coded based on the value of their correlation peak. **(B)** The same graphs obtained after removing electrodes without any neuron and reweighting based on the structural distance. The refined FC graph shows a better coherence with the network topology.

Functional connectivity graphs were computed for the two neuronal cultures using the cross-correlation algorithm, followed by spatio-temporal filtering and thresholding, as described in Section 2.4. The resulting FC maps are provided in Figure 6A where functional links are color-coded based on the value of the cross-correlation peak. Both graphs—even after selecting only the statistically significant connections—present a substantial number of long-range links. According to what is suggested in Section 2.5, electrodes without any neuron in their recording area were first removed from the functional graphs. This allowed to reduce the number of functional links by 22% in the case of Chip-253 and by 37% for Chip-250.

**Figure 7:**
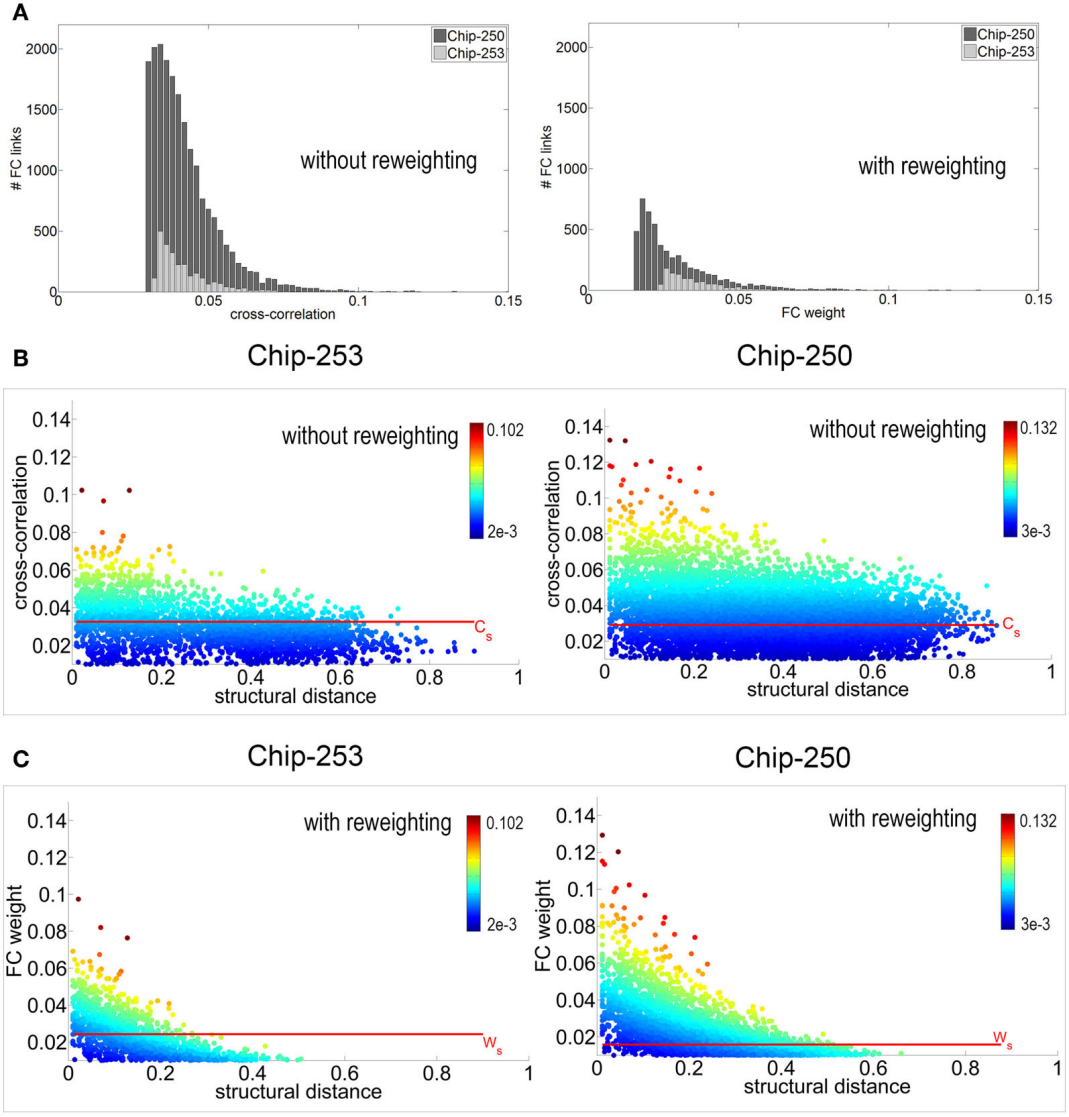
**(A)** Distribution of functional weights before and after reweighting. **(B)** Weight vs. distance plot *before* reweighting. Points are color-coded with a heat colormap based on the correlation value. The significance threshold *C*_*s*_ is computed using a statistical test with *p*-value *p* = 0.05. **(C)** Weight vs. distance plot *after* reweighting. Thanks to the structural prior, the new significance threshold *W*_*s*_ allows to select functional connections that are more coherent with the structural topology of the network.

The proposed reweighting method was then applied to investigate which of the remaining functional links actually relied on a structural path. It should be noted that the use of the shortest path between pairs of electrodes is a choice that favors shorter structural connections which are more likely to be direct or, in general, morphologically plausible. Although this does not guarantee that the chosen path is the one actually active, we assume that—statistically—minimal paths are the most probable ones (Vincent et al., 2013). Figure 7A shows the distribution of functional weights with and without the graph refinement. A substantial decrease in the number of functional connections can be observed as a result of incorporating the structural information into the initial FC estimates. To better highlight this effect, weight vs. distance scatter plots are also provided in Figure 7B. The plots are referred to the initial functional links obtained after spatio-temporal filtering and after removing the electrodes without neurons. Points are color-coded using a heat colormap based on the link's cross-correlation. It can be observed that higher correlations correspond to shorter paths and that an increase in the structural distance weakens the corresponding functional correlation. The red line represents the significance threshold *C*_*s*_ obtained from the statistical test. Although applying this threshold would allow to discard 37.1% of connections for Chip-250 and 68.3% for Chip-253, the result still presents many long-range functional links.

Unfortunately, after the statistical significance test, there is no easy or intuitive way for neuroscientists to make a further distinction between relevant and spurious connections. More conservative estimates of functional connectivity are sometimes provided by ranking the estimated links according to their cross-correlation values and selecting the strongest *K* (e.g., *K* = 100, Maccione et al., 2012). However, the problem of determining a satisfactory value for *K* still remains. Ideally, we would like to threshold the FC graph in order to privilege short-range connections while penalizing long-range ones. At the same time, we want to allow the selection of links with substantial correlation even on long distances.

The proposed reweighting formula allows to meet these requirements, as shown by the scatter plots of Figure 7C. Points are plotted according to their new weight but they maintain the initial color of Figure 7B. This allows to highlight how the significance threshold applied to the reweighted graph can effectively discard functional links that are too distant, even when they have significant correlation. Figure 6B shows the refined functional graphs for the two networks under study obtained after applying the significance threshold *W*_*s*_. Results present an overall decrease in the number of FC links by 86.5% for Chip-253 and 83.7% for Chip-250 with respect to the initial cross-correlation estimates. As opposed to the functional connectivity graph shown in Figure 6A, the introduced strategy automatically selects functional connections that are more coherent with the structural topology of the network. This can be more evidently observed in the case of Chip-253, where the culture presents a clear clustering into two subnetworks that are almost completely separated from each other. Nevertheless, the initial estimate of the FC graph—relying only on the electrophysiological signals—included a massive amount of links connecting the two subnetworks. Thanks to the reweighting process we are capable of filtering such FC links, retaining only the ones being coherent with the structural prior or showing a substantially strong correlation. The introduced reweighting formula allows to penalize correlation with distance while modulating the contribution of the structural prior, based on the amount of evidence on the functional co-activation. Thanks to the proposed formulation, lower correlations are more strongly penalized with distance—thus imposing stronger structural prior—whereas higher correlations are less influenced by the neuronal displacement as the functional evidence prevails. This provides a more conservative estimate of functional connectivity, as compared to other weighting functions. For instance, in case of a negative exponential function [i.e., *C*_*P*_(*x*, *y*) *e*^−*d*_*xy*_^], at a given distance, higher correlations would be more penalized than smaller ones. This would imply a substantial influence of the structural prior, even in presence of strong evidence of functional co-activation. On the contrary, with our approach structural and functional information are combined preserving the contribution of clear functional observations without imposing a too strong structural prior. Table 2 summarizes the quantitative results on the structural and functional analysis of the considered datasets.

**Table 2:** **Quantitative structural/functional information**.

		**Chip-253**	**Chip-250**
Structure	Neurons	1312	1152
	SC links	5570	7808
Function	FC links (initial estimate)	4085	16290
	FC links (above threshold *C*_*s*_)	1294	9788
	Discarded FC links (above threshold *C*_*s*_ w.r.t. initial estimate)	2791 (68.3%)	6052 (37.1%)
Structure + Function	FC links (neurons only)	3187	10296
	FC links (neurons only, above threshold *C*_*s*_)	1041	6258
	FC links (final estimate: reweighted, above threshold *W*_*s*_)	553	2654
	Discarded FC links (reweighted w.r.t. initial estimate)	3532 (86.5%)	13636 (83.7%)

### 3.2. Relevant features for functional analysis

**Figure 8:**
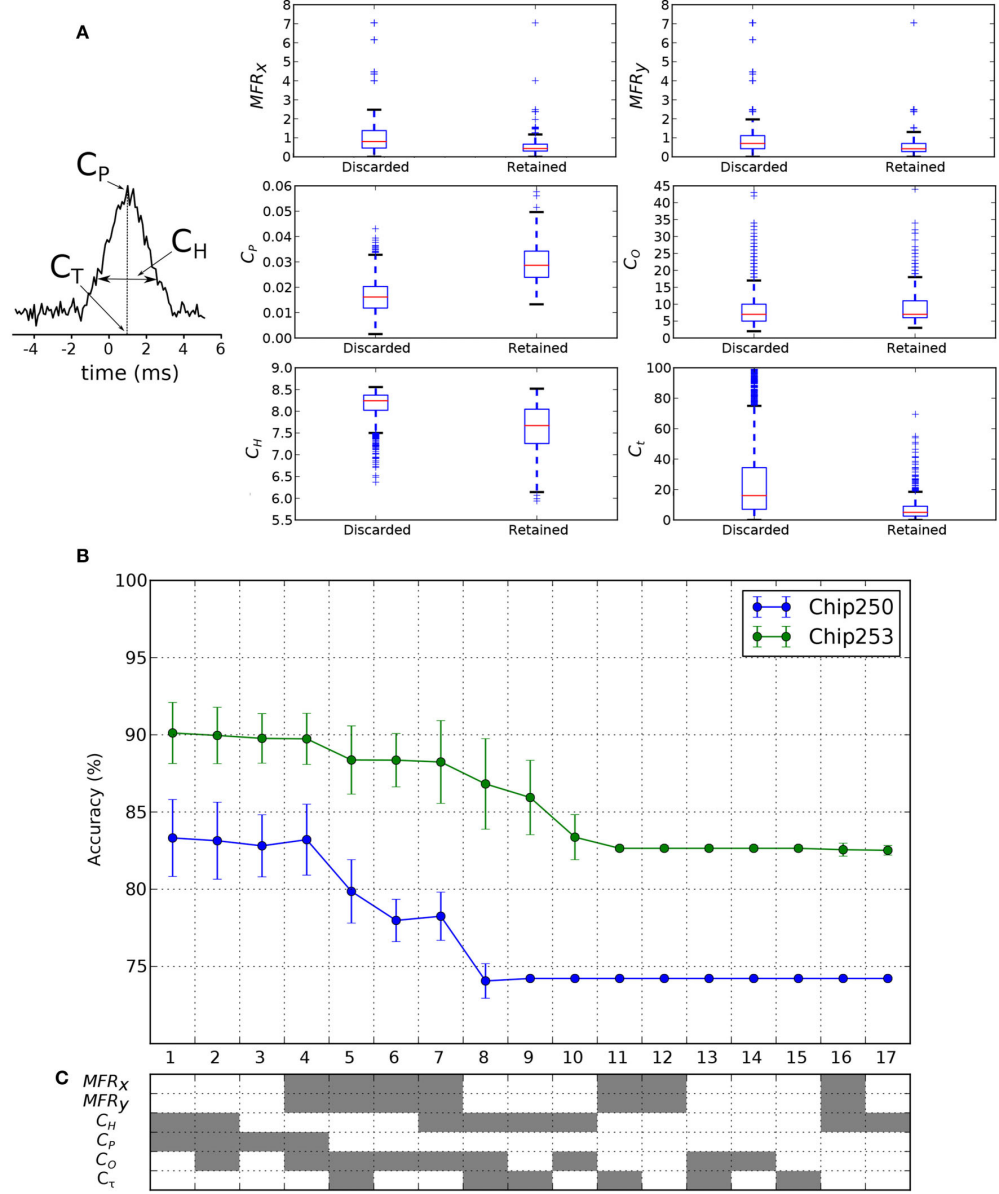
**Classification features and results**. **(A)** Cross-correlation features used in the classification process. The peak of cross-correlation *C*_*P*_, its peak time *C*_τ_ and the corresponding spread *C*_*H*_ are considered. **(B)** Box plots of the feature distributions in the discarded and retained link classes. The retained class shows significantly higher *C*_*P*_ and lower *C*_*H*_. **(C)** The classification accuracy in Chip-250 and Chip-253 shows a similar ranking, from 1 (highest) to 17 (lowest), as function of the considered features. The used combinations of features are encoded in gray below the plot.

We want to investigate if the functional features of discarded/retained FC links can suggest new hypotheses on the way neurons functionally interact. To this purpose, we want to assess and compare the relevance of commonly used functional features for the classification of FC links belonging to the two classes (discarded or retained links, according to the structural prior). Some of these functional features are directly computed from the cross-correlation function. The cross-correlation peak *C*_*P*_, its time lag *C*_τ_ and the spread *C*_*H*_ are reported in Figure 8A. Then, as indicated in Section 2.6, *C*_*O*_ is computed from *C*_*P*_, *MFR*_*x*_, and *MFR*_*y*_. To gain some insights on the potential discriminative power of each feature, we first compared their distributions across the two classes of discarded/retained connections. Results are shown in the box plots of Figure 8B. As intuitively expected, the distribution of correlation peaks *C*_*P*_ is significantly different from one class to the other, as this feature is directly involved in the FC graph estimation. The features *C*_*H*_ and *C*_τ_ show smaller values in the retained dataset, indicating that the reweighting procedure was effective in selecting cross-correlation functions with reduced spreads and time lags. The latter result shows that the retained features actually correspond to more reliable (i.e., lower entropy) and more physiological (i.e., the peak is closer to the integration time of synaptic events) functional links. Then, subsets of the considered features were used to train and test the SVM classifier. The corresponding ranked accuracies (mean ± std on 10-fold cross-validation) are reported in Figure 8C and confirm that *C*_*P*_ is the most significant feature (x-axis: 1–4). Interestingly, Figure 8C shows that when *C*_*P*_ is removed from the tested features (x-axis: 5 on) a reasonable level of accuracy can still be achieved by the linear SVM. This holds true for different combinations of features (Figure 8C, x-axis: 5–7 for Chip-250; x-axis: 5–9 for Chip-253) indicating that other features are also informative for discriminating retained from filtered links. Specifically, the mean firing rates (*MFR*_*x*_, *MFR*_*y*_) can be alternatively combined with the *C*_*O*_, *C*_*H*_, and *C*_τ_ features still yielding a good discriminative power. Finally, the computed accuracies reach a plateau (Figure 8C, x-axis: 8–17 for Chip-250; x-axis: 11–17 for Chip-253) that corresponds to the noise level of the classifier (i.e., the chance of a random classification). Indeed, based on Table 2, the noise level (*ACC*_η_) was computed analytically^2^ and matched with the corresponding plateaus (Chip-250: *ACC*_η_ = 74.2%; Chip-253: *ACC*_η_ = 82.6%). The plateau region is characterized by single as well as subgroups of features (e.g., *C*_*O*_, *C*_τ_, {*MFR*_*x*_, *MFR*_*y*_} and *C*_*H*_) that are ineffective for discriminating the retained from the discarded links. In conclusion, apart from *C*_*P*_, we found that different combinations of features can also be effective in discriminating the retained from the discarded links thus motivating the development of alternative algorithms that incorporate this information to improve the detection of structurally-coherent functional connectivity maps.

### 3.3. General discussion and perspectives

The study of the relationship between structure and function at the mesoscale, taking advantage of multielectrode arrays and fluorescence microscopy, has to face two important issues: (i) a limited optical resolution for the structural description, and (ii) the need for resolving single neurons from extracellular recordings on the functional aspect. Knowing this limitation in resolution, a central concept in our approach is to take advantage from the combination of *partial* descriptions of structure and function to generate a refined estimate of the network activity. Furthermore, to place our study in the best conditions to properly validate the proposed ideas, we adopt low-density cell cultures and high-density MEAs. As a matter of fact, this combination allows to typically record one single-unit from each electrode of the 4096-array (this holds for about 90% of the electrodes). This settings allows to minimize the shared variance given by the potential recording of many neurons from a single electrode. On the other side, the issue of cross-talk that might be given by the recording of the same neuron from many nearby electrodes is minimized by the low-density culture condition and by the electrode density of the CMOS-MEA that provides a small inter-electrode separation of 21 μm. Finally, we deliberately use low-density cell cultures as they enable to validate the proposed framework allowing to identify single neurons and estimate their connectivity within large neuronal networks. However, in principle, the basic concepts of the presented methodology might also be applied to denser cell cultures, to *ex vivo* brain tissue preparations or even to *in vivo* experimental studies on subsets of neural populations expressing fluorescent markers. This would be feasible upon the adoption of sufficiently high-resolution microscopy and recording techniques. For instance, having higher plating densities would imply a much larger number of structural connections. In this case, the problem complexity would lie in a correct and reliable encoding of the local neuritic architecture. As the proposed local directional features are capable to deal with complex structures showing many crossing and branching neurites, despite the increase in the computational load, it would be possible to apply the same feature-based analysis. The heat kernel propagation could then be used to estimate the structural connectivity even in such denser neuronal preparations.

## 4. Conclusions

Although functional analysis at the mesoscale is typically carried out with coarse or absent structural information, thanks to the HD-MEA technology and to the proposed structural analysis, it was possible to move a step forward relating network-scale functional and structural data at cellular resolution.

In this paper, we presented a computational framework capable of estimating structural and functional connectivity graphs from immunofluorescence images and electrophysiological recordings of *in vitro* neuronal networks cultured on HD-MEAs. As functional correlation and structural distance have been shown to be related both theoretically and in different experimental conditions (Hirase et al., 2001; Shlens et al., 2006; Kriener et al., 2009; Vincent et al., 2013), we introduced a reweighting strategy that allows to refine correlation-based measures of functional connectivity using the acquired structural prior. Such refined estimates were then used to investigate the role of different functional features in actual neuronal interactions. Our analysis showed that the combination of structure and function allows to obtain reliable functional connectivity graphs that are more coherent with the network topology and, as a consequence, with the known distance-dependent neuronal behavior. The classification results also allowed to reveal how different combinations of features can be more informative than others when targeting the detection of correlated functional activities.

Thanks to the cellular resolution offered by the HD-MEA technology, the proposed approach allowed, for the first time, to obtain a full characterization of the structural and functional connectivity at the mesoscale with a granularity of the single cell. This first attempt in combining structure and function at this level paves the way toward a deeper understanding of the low-level functions of complex circuits from which higher-level brain behaviors emerge.

Further investigation will target the analysis of more advanced reweighting techniques, based on a probabilistic modeling of the relationship between cross-correlation and structural distance or other relevant features of the structural graph. Complementary future work will address the analysis of dissociated networks with selective immunofluorescence staining to separate the contributions of inhibitory and excitatory subnetworks and study their structure-function interplay.

### Conflict of interest statement

The authors declare that the research was conducted in the absence of any commercial or financial relationships that could be construed as a potential conflict of interest.

## Footnotes

^1^Imaging and electrophysiological datasets will be available on CARMEN (https://portal.carmen.org.uk) upon request of access credentials to the corresponding author.

^2^*ACC*_η_ = *max*(*nDIS*, *nRET*)/(*nDIS* + *nRET*) with nDIS/nRET corresponding to the cardinalities of the discarded/retained data sets.
